# How to Combat Gram-Negative Bacteria Using Antimicrobial Peptides: A Challenge or an Unattainable Goal?

**DOI:** 10.3390/antibiotics10121499

**Published:** 2021-12-07

**Authors:** Adriana Barreto-Santamaría, Gabriela Arévalo-Pinzón, Manuel A. Patarroyo, Manuel E. Patarroyo

**Affiliations:** 1 Receptor-Ligand Department, Fundación Instituto de Inmunología de Colombia (FIDIC), Carrera 50#26-20, Bogotá 111321, Colombia; adrianasantamaria10@gmail.com; 2 Science Faculty, Universidad de Ciencias Aplicadas y Ambientales (U.D.C.A), Calle 222#55-37, Bogotá 111166, Colombia; 3 Biology Department, Science Faculty, Universidad Antonio Nariño, Carrera 3 Este # 47 A-15, Bogotá 110221, Colombia; garevalo78@uan.edu.co; 4 Molecular Biology and Immunology Department, Fundación Instituto de Inmunología de Colombia (FIDIC), Carrera 50#26-20, Bogotá 111321, Colombia; mapatarr.fidic@gmail.com; 5 School of Medicine, Universidad Nacional de Colombia, Carrera 45#26-85, Bogotá 111321, Colombia; 6 Health Sciences Division, Main Campus, Universidad Santo Tomás, Carrera 9#51-11, Bogotá 110231, Colombia

**Keywords:** Gram-negative bacteria, haemolysis, minimal haemolytic concentration (MHC), minimal inhibitory concentration (MIC), clinical trial, antimicrobial resistance

## Abstract

Antimicrobial peptides (AMPs) represent a promising and effective alternative for combating pathogens, having some advantages compared to conventional antibiotics. However, AMPs must also contend with complex and specialised Gram-negative bacteria envelops. The variety of lipopolysaccharide and phospholipid composition in Gram-negative bacteria strains and species are decisive characteristics regarding their susceptibility or resistance to AMPs. Such biological and structural barriers have created delays in tuning AMPs to deal with Gram-negative bacteria. This becomes even more acute because little is known about the interaction AMP–Gram-negative bacteria and/or AMPs’ physicochemical characteristics, which could lead to obtaining selective molecules against Gram-negative bacteria. As a consequence, available AMPs usually have highly associated haemolytic and/or cytotoxic activity. Only one AMP has so far been FDA approved and another two are currently in clinical trials against Gram-negative bacteria. Such a pessimistic panorama suggests that efforts should be concentrated on the search for new molecules, designs and strategies for combating infection caused by this type of microorganism. This review has therefore been aimed at describing the currently available AMPs for combating Gram-negative bacteria, exploring the characteristics of these bacteria’s cell envelop hampering the development of new AMPs, and offers a perspective regarding the challenges for designing new AMPs against Gram-negative bacteria.

## 1. Introduction

Infectious diseases represent one of the major causes of death worldwide; lower respiratory route infections (mainly caused by viruses and bacteria) are the most deadly transmissible diseases, having caused around 2.6 million deaths worldwide in 2019 [1]. Gram-negative bacteria head the list of pathogens having the highest resistance indices of all bacteria threatening human health; they thus head the priority pathogens list for research and development (R&D) for new antibiotics. The most critical group on this list includes *Acinetobacter*, *Pseudomonas* and various members of Enterobacteriaceae (including *Klebsiella*, *Escherichia coli*, *Serratia* and *Proteus*) which could cause serious and/or mortal infections, such as bloodstream infections and pneumonia [2].

In spite of great efforts having been made to combat these bacteria, the development of new antibiotics has become substantially hampered by their high mutation rates and the rapid appearance of resistance to antibiotics, making them financially unattractive for the pharmaceutical industry [3,4,5].

Reduced research efforts and funding in the field of antimicrobials was mainly focused on multidrug-resistant (MDR) Gram-positive bacteria more than two decades ago, as they represented the greatest threat to health, meaning that research into new antimicrobials against Gram-negative bacteria lagged behind [6,7]. Consequently, only two totally new antibiotics acting exclusively against Gram-positive bacteria have been approved during the last two decades: The oxazolidinone family (linezolid and tedizolid) and daptomycin (a cyclic lipopeptide) [8]. Such a low antibiotic production rate has led to exploring AMPs as a hopeful alternative for combating Gram-negative bacteria. AMPs with membranolytic action have drawn attention because such a mechanism enables rapid action against pathogens, thereby providing them with fewer opportunities to resist [9,10,11,12]. However, despite their enormous advantages documented since their discovery, colistin has been the only AMP which has achieved recognition as being suitable for managing MDR Gram-negative pathogens is colistin [13].

AMPs do have some weaknesses, such as their toxic action against mammalian cells, giving them a low therapeutic index (TI), high production costs associated with their length and poor stability and bioavailability in in vivo conditions. Such drawbacks have led to exploring chemical modifications and design strategies for obtaining synthetic AMPs having better therapeutic properties [14,15,16,17]. However, design, particularly against Gram-negative bacteria, represents an arduous, high complexity process, frequently leading to obtaining peptides having little or no activity or poor selectivity, thus being far from fulfilling a new candidate AMP’s initial expectation [18,19]. One cause of such a situation concerns Gram-negative cell envelops representing a biological challenge for peptide molecules. Developing new AMPs against Gram-negative bacteria should thus concentrate on overcoming this type of bacteria’s main barriers and broadening knowledge regarding AMP–bacteria interactions and AMPs’ physicochemical properties defining such interactions. The first part of this review describes the main AMPs in clinical stages or approved by the FDA against Gram-negative bacteria. Such a meagre panorama regarding new AMP development suggested a review of the Gram-negative bacterial membrane components representing a challenge to AMP action for the second part. Critical focal points are proposed for anti-Gram-negative AMP design and development.

## 2. Which AMPs Are Available for Combating Gram-Negative Bacteria?

**Figure 1:**
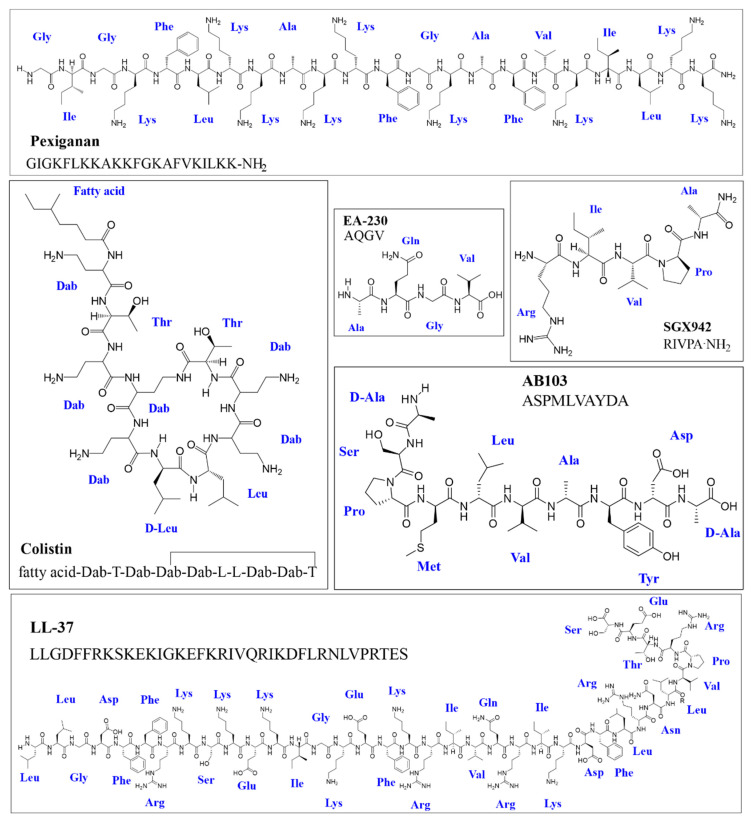
FDA-approved AMP structure and that of antibacterial or immunomodulatory peptides in development against Gram-negative bacteria. Colistin, pexiganan and LL-37 are large molecules having direct antibacterial activity and a membranolytic mechanism of action. Peptides EA-230, AB-103 and SGX-942 do not have direct antibacterial activity; however, they do have an immunomodulator effect which helps resolve Gram-negative bacterial infection in vivo. A bond between Dab’s side chain and Thr’s carboxyl terminal forms the cycle. PerkinElmer ChemDraw Professional 16.0.1.4 molecule editor was used for drawing the structures.

Around 84% of the natural AMPs in the antimicrobial peptide database (APD3) have been registered as being antibacterial [20], having anti-Gram-negative, Gram-negative activity or against both of them. However, most AMPs in clinical development or FDA approved have been shown to lack potency against Gram-negative bacteria [7,14]. Only one of the seven antibacterial peptides (some natural, others synthetic) approved by the FDA to date [21] is active against Gram-negative bacteria. Moreover, just five of the 19 antimicrobial or immunomodulatory peptides currently in advanced clinical development phases are designed for treating infection caused by Gram-negative bacteria [16], two having direct membranolytic effect. So, only three AMPs (one FDA approved and two in development) and three immunomodulatory peptides will be available for treating Gram-negative bacteria infections (Figure 1 and Table 1).

AMPs which have been approved or are in clinical development have had 1.7 to 16 µM MIC values against Gram-negative bacteria (Table 1). It is worth noting that although such values can act as reference regarding activity, determining MIC is influenced by many experimental factors such as the ion concentration in the medium used, initial bacterial inoculum and pH [37,38,39]. AMPs in clinical development (colistin, pexiganan and LL-37) have haemolytic activity at their MIC so their administration route has been limited to just topical use as their toxicity prohibits systemic application. Such toxicity was evident during their in vitro evaluation in haemolysis assays showing their ability to lyse erythrocytes at MIC, i.e., poor microorganism selectivity (low TI) (Table 1).

1. Colistin or polymyxin (natural): Colistin is a cyclic decapeptide incorporating a fatty acid chain at the N-terminus with six Dab (an unusual amino acid called L-2,4-diaminobutyric acid) (Figure 1). It has a +5 net charge at physiological pH and has direct membranolytic antibacterial activity and potent anti-endotoxin activity due to its direct interaction with lipopolysaccharide (LPS) [40,41]. Colistin was discovered in 1947, FDA approved and used for more than a decade (since 1960); however, in the 1970s it became overlooked due to its adverse effects. Nonetheless, due to increasing preoccupation regarding the lack of effective new antimicrobials, its controlled use has been reconsidered and is now regarded as a safe drug [42]. Colistin is currently on the World Health Organisation’s model list of essential medicines (21st list, 2019) as a last-resort antibiotic and is the only AMP approved that is selective against Gram-negative bacteria [43].
2. Pexiganan or MSI 78 (designed): Pexiganan was the first animal-derived peptide (isolated from frog skin) to reach phase III clinical studies. It is a 22 residue-long molecule which was synthesised with the amide group at the C-terminus extreme and is currently in phase III clinical studies (Figure 1) [19]. This molecule disrupts the membrane via toroidal-type pore formation thereby enabling it to have a bactericidal effect against Gram-negative and Gram-positive bacteria [44]. The best results have been obtained with this molecule regarding a cream for treating diabetic foot infections [27,28]; however, it is not FDA approved because it has not been shown to have advantages over currently available agents in terms of safety and therapeutic effectiveness [19].
3. LL-37 (natural): LL-37 is a natural human cathelicidin hCAP18-derived from a 37 amino acid-long peptide (Figure 1). LL-37 is currently in phase II trials regarding its topical use for treating bacterial colonisation, inflammation response and diabetic foot ulcer healing rate (NCT04098562). This peptide has been one of the most extensively studied AMPs due to its diverse biological properties as it has an LPS neutralising, immunomodulatory and chemiotactic effect in addition to having direct antimicrobial activity against a broad range of microorganisms by pore formation [30,45]. However, its toxicity has led to problems regarding protease stability and production costs associated with its length; multiple shorter derivatives have been evaluated, demonstrating improved properties which would probably have greater therapeutic scope than LL-37 [46].
4. Other AMPs in clinical stage trials: Broad-spectrum AMPs (i.e., those derived from melittin and protamine, 29 amino acid-long melimine (ACTRN12613000369729) and its 15 amino acid-long derivative mel4 (ACTRN1261500072556)) are being evaluated in clinical phase as alternatives for preventing Gram-negative and Gram-positive microbial colonisation of contact lenses [32,33]. Engineered cationic antimicrobial peptide (eCAP) WLBU2 (PLG0206) is in phase I clinical trials (ACTRN12618001920280); it has been projected for the treatment of prosthetic joint infections due to its anti-Gram-positive bacteria activity [34]. However, this broad-spectrum peptide could be useful for treating Gram-negative bacteria infections such as *P. aeruginosa* and other multi-resistant bacteria [39]. WLBU2 has proven to be superior to natural peptides such as colistin and LL-37 due to it having less potential for inducing resistance and greater stability in in vivo conditions [36,37]. In spite of this, WLBU2′s poor selectivity continues to be a limiting factor [39].
5. Anti-Gram-negative AMPs with an uncertain therapeutic future: A peptide may have promising characteristics in in vitro conditions, or even in animal model trials; however, this does not guarantee success along the long road to approval by the FDA [14,47]. AMPs seeming to be a promising alternative for combating Gram-negative bacterial infections have sometimes had unexpected adverse effects (AF) during more advanced stages. For example, it emerged during phase II and III studies that the use of talactoferrin (TLF, rhLF) for treating severe sepsis was prejudicial, as mortality was higher in the group of patients receiving this AMP than in the control group [48]. Another example concerns the protegrin 1 derivate called murepavadin (POL7080); its phase III study regarding treatment of *Pseudomonas aeruginosa*-related hospital-acquired pneumonia was terminated before time due to safety problems connected with renal failure (ClinicalTrials.gov Identifier: NCT03582007). Another scenario deals with the difficulty of demonstrating promising results during the clinical phase suggested by in vitro assays; a clear example of this would be iseganan (another protegrin 1 derivative) which did not prove effective during phase III trials for preventing ventilator-associated pneumonia or as mouthwash for reducing mucositis and stomatitis (oral mucositis) in chemotherapy patients via oral administration [49,50].

Such scenarios, along with the accelerated development of antimicrobial resistance to peptides like colistin or murepavadin, have led to underestimating AMPs’ usefulness in combating Gram-negative bacteria [51]. However, AMPs continue being the focal point of study for developing new therapeutic agents against this bacterial group regarding the enormous problem concerning resistance and the lack of alternatives for combating it.

Peptides from the main AMP families currently in in vitro studies will surely lead to highly competitive therapeutic candidates for combating Gram-negative bacteria. Such AMPs have been obtained by modifying natural AMPs to optimise their activity and selectivity. Table 2 gives some examples of these peptides. However, new candidate research should not be limited to the exploration of classic families; other sources and approaches must be adopted to develop a powerful arsenal of new, effective molecules against Gram-negative bacteria.

An AMP must have low toxicity against erythrocytes to be of interest regarding its systemic application, meaning that measuring haemolytic activity could be useful as a first filter for selecting therapeutic candidates [59]. It should be stressed that determining haemolytic activity is influenced by experimental factors such as the amount of erythrocytes used [60]. Furthermore, as erythrocytes are specialised cells and lack organelles, they represent an extremely limited model for representing mammalian cells [60]. So, the search for new candidates having high selectivity from the laboratory stage onwards [14] and the development of new in vitro techniques addressing cell selectivity evaluation more fully [60] must become a priority.

## 3. Cell Envelope: Gram-Negative Bacteria’s Lipid Rampart

A peptide’s antibacterial activity partly depends on the cell envelope components of the bacterium in question. A peptide’s physicochemical properties, along with such components, will define the peptide-bacteria interactions giving rise to the antibacterial effect. Gram-negative cell envelops represent a relevant barrier making them intrinsically resistant to many antibiotics, including AMPs [61].

### 3.1. Outer Membrane (OM)

**Figure 2:**
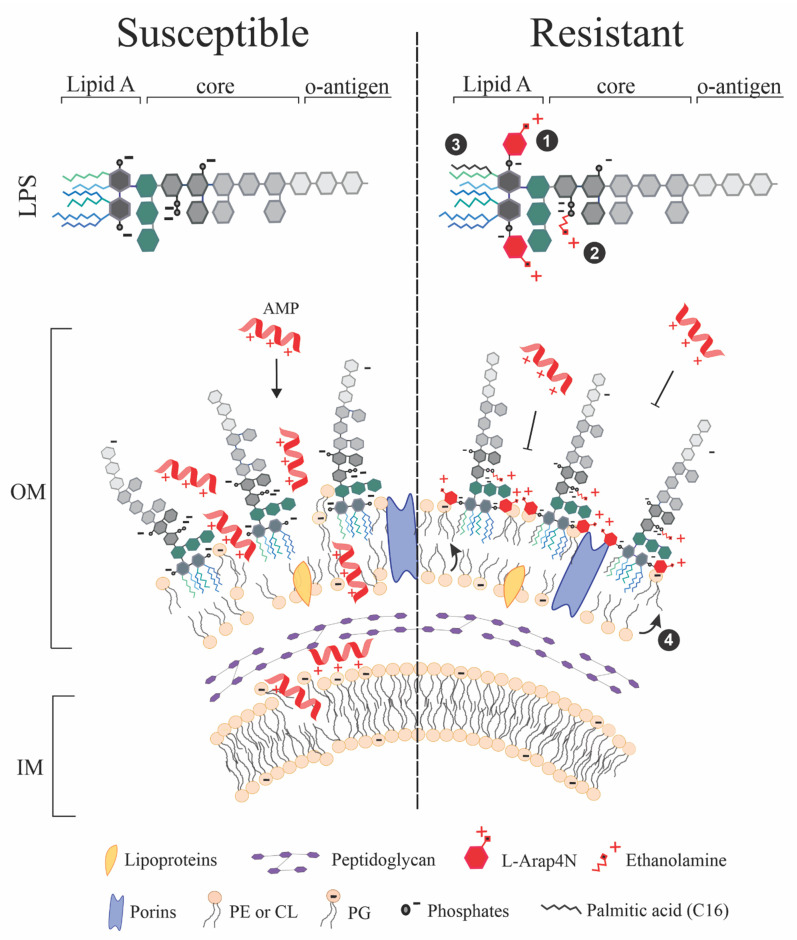
AMP-susceptible Gram-negative bacteria cell envelop compared to that of Gram-positive bacteria resistant ones. LPS-rich negatively charged outer membrane (OM) is prone to interact with cationic AMPs (left), whilst LPS altered by (1) ArnT-mediated 4′-phosphate (negatively charged) being replaced by 4-amino-4-deoxy-L-arabinopyranose (L-Arap4N) (positively charged), or (2) EptB-mediated ethanolamine molecules (positively charged) added to the diphosphates (negatively charged) lead to electrostatic repulsion with cationic AMP amino acids, making bacteria resistant to them. (3) LPS lipid A palmitoylation (i.e., adding palmitic acid, usually transferred from PG or PE) and (4) PG palmitoylation (i.e., adding palmitic acid, usually transferred from PE) leads to a better hydrophobic barrier for AMP penetration. Once AMPs reach the inner membrane (IM), negative charge density becomes a decisive factor for its membranolytic action, as bacteria having greater charge density are usually more susceptible to these AMPs.

**Figure 3:**
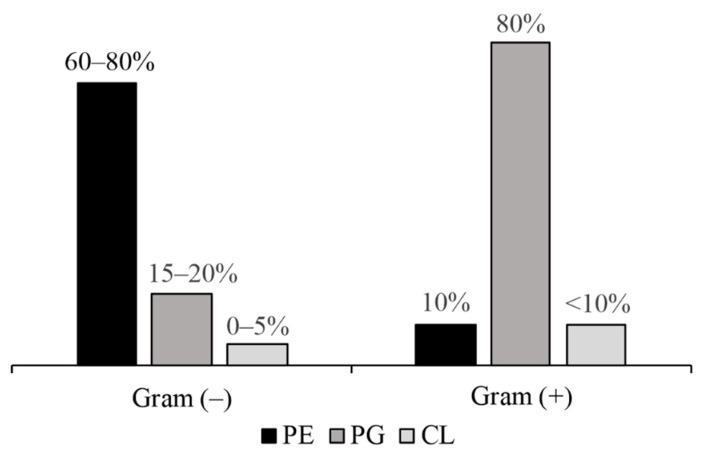
Gram-negative and Gram-positive bacterial membranes’ phospholipid composition. Phosphatidylethanolamine (PE), phosphatidylglycerol (PG) and cardiolipin (CL) percentages are shown. Compiled from data reported in [78,83].

The outer membrane has been highlighted as determinant regarding Gram-negative bacteria sensitivity or resistance to antibiotics since changes in its composition condition different species’ sensitivity to them [6]. Gram-negative bacteria outer membrane has a phospholipid (PL) bilayer which is similar to that of the internal membrane (Figure 2 and Figure 3) but has a less robust and asymmetric composition due to having porins and LPS on the external face and lipoproteins on the internal face. Porins are proteins enabling the passage of small hydrophilic molecules (less than 600 Da) through the external membrane and differences in composition and the presence of these proteins condition Gram-negative bacteria sensitivity to some antibiotics [6]. As AMPs have greater molecular weight they cannot pass through porins [62], meaning that they must permeabilise the external membrane bilayer, thereby opening a passage for gaining access to deeper targets, such as the internal membrane [63] or intracellular targets [64].

LPS is the first contact site for AMPs; Gram-negative bacteria resistance or susceptibility to some AMPs is due to changes in LPS charge [65,66,67]. LPS has a basic structure containing an acylated D-glucosamine bisphosphate main chain in N and O (called lipid A), a central polysaccharide and a variable terminal polysaccharide called antigen O. AMP activity and selectivity for certain bacterial species has been related to their affinity for lipid A where electrostatic and hydrophobic interactions prevail [68]. Changes in AMP-resistant bacteria are usually related to lipid A charge and structure and are enzyme-mediated. For example, 4′-phosphate (negatively charged) in lipid A is replaced by 4-amino-4-deoxy-L-arabinopyranose (L-Arap4N) (positively charged) in an ArnT-mediated process or ethanolamine molecules (positively charged) are added to the diphosphates (negatively charged) in an EptB-mediated process; these changes lead to electrostatic repulsion with cationic AMP amino acids thereby making bacteria resistant to AMPs (Figure 2) [65,69]. Such changes explain why AMPs like polymyxin B are less active against species such as *Proteus mirabilis* which usually has less negatively charged LPS than most *E. coli* strains [65] or why some *E. coli*, *Klebsiella pneumoniae* or *P. aeruginosa* strains are resistant and others sensitive to polymyxins-B or -E [69,70,71].

Increased amounts of lipid A acylated chains (palmitoylation) due to transfer from PL phosphatidylethanolamine (PE) or phosphatidylglycerol (PG), and even from PE to PG, may also reduce Gram-negative bacteria’s susceptibility to α-helicoidal, amphipathic AMPs due to an increase in the hydrophobic barrier, thereby preventing their penetration [66,67].

As some enzymes are involved in the development of Gram-negative bacteria’s resistance, it has been proposed that enzymatic inhibitors could be complementary therapeutic targets for AMPs due to their adjuvant effect [72]. For example, using metabolites such as ent-beyerane diterpenes which bind and block ArnT action regarding *P. aeruginosa* or non-antimicrobial macrolides (like eukaryotic phosphatase inhibitors) could act as colistin adjuvants, making it effective against strains which were originally resistant to it [71,73]. In this context, Gram-negative bacteria’s PhoPQ two-component regulatory system should be taken into account as a complementary target of action too, due to its fundamental role in external membrane modification and global regulatory role in antibiotic susceptibility, physiology, stress adaptation and virulence in bacteria [66,74].

LPS has complications in vivo related to an individual’s immune response. Gram-negative cell death and division trigger LPS release to the environment, thereby stimulating the activation of an organism’s immune cell response and could lead to sepsis and septic shock. Consequently, an agent causing these microorganisms’ death in serious infections will not be sufficient for guaranteeing patient recovery. LPS neutralisation is a second task that must be undertaken regarding the search for anti-Gram-negative antimicrobials. AMPs thus represent an ideal neutralisation strategy [75] and could thereby represent more suitable molecules than other antibiotics for treating Gram-negative infections or could be used in combination with conventional antibiotics due to their ability to neutralise released LPS, either by non-specific electrostatic interactions with LPS, or through an immunomodulatory effect exercised by some AMPs [34,35,67].

### 3.2. Inner Membrane

Following initial interaction with LPS and external membrane permeabilisation, AMPs reach the cytoplasmatic membrane (where electrostatic AMP–membrane interaction enables peptide accumulation on the PL bilayer); reaching such a perforation threshold produces collapse and the membrane breaks causing bacterial death [76,77]. Gram-negative bacteria’s internal membrane mainly consists of zwitterionic PL PE and negatively charged PL PG to a lesser extent (Figure 3) [78]. Negative charge density on the cytoplasmatic membrane could be determinant for AMP action. Gram-positive bacteria have greater negative charge density due to having high PG content (Figure 3) which could facilitate some AMPs’ action against them and could explain AMPs’ action against both types of bacteria. An example of this would be alterations in PG/PE liposomes due to membranolytic AMPs having preferential PG interaction and capture [79].

Some AMPs preferentially acting against Gram-negative bacteria seem to interact more strongly with PE than PG, as can be seen with cationic peptide 35,409 which acts against Gram-negative *E. coli*. This peptide causes greater disruption in PE liposomes than in PG or PE/PG ones [80]. Preferential interaction with PE could explain such preference against Gram-negative bacteria and could be related to collateral activity against eukaryotic cells which are also PE-rich [80,81,82]. Such Gram-negative bacterial membrane PL similarity with eukaryote cell membrane poses an additional challenge regarding research into AMPs acting against Gram-negative bacteria and places demands on researchers to refine AMP design strategies.

## 4. Engineering AMPs against Gram-Negative Bacteria

It is known that a significant group of antibacterial peptides are cationic and have α-helix structure thereby promoting their membranolytic action against Gram-negative and Gram-positive bacterial cells [84]; however, little is known about the physicochemical characteristics determining activity against such bacterial groups. No evident differences regarding the properties of AMPs acting against Gram-negative bacteria and those acting against Gram-positive bacteria were found when comparing the length, net charge, hydrophobicity, hydrophobic moment, isoelectric point and the most frequently occurring amino acids for more than 17,000 natural and synthetic peptides in the Database of Antimicrobial Activity and Structure of Peptides (DBAASP) (https://dbaasp.org/statistics/compositional, accessed on 1 December 2021) [85,86].

However, some investigations have suggested certain characteristics’ relevance. For example, an analysis of AMP evolution in molluscs and crustaceans has suggested some sequence motifs; these have been maintained in such animals’ AMPs as time has elapsed and are related to their activity against Gram-negative or Gram-positive bacteria. Regarding AMPs acting against Gram-positive bacteria, the presence and relevance of cysteines and sequence motifs mediating these AMPs’ interaction with lipid II (bacterial wall synthesis precursor in Gram-positive bacteria) in molluscs’ defensins has been highlighted. The motifs stressed as being relevant for selectivity against Gram-positive were: The γ-core (containing Gly-X-Cys sequence), the very conserved N-terminal sequence consisting of GFGC or ATCDL (usually followed by the ATCDL(10) CAXHC X(6) GGYC X(5) CVCRN motif) and the GYC motif [87].

Regarding AMPs against Gram-negative bacteria, evolutionary analysis of crustacean defensins has indicated that the conserved LPS binding motif consists of a negative amino acid and six positively charged residues interacting with LPS lipid A negative charges (E, K, K/R, K, K/R, K/R, K/R) [87]. The design of a selective AMP against Gram-negative bacteria should thus theoretically contain the LPS binding motif and avoid the lipid II binding motif; however, designing Gram-negative selective AMPs is more complicated than that.

As negative charge density is greater on Gram-positive than Gram-negative cytoplasmic membrane (Figure 3) [78,83], differences at this point could be related to AMP selectivity for a type of bacteria or its indiscriminate activity regarding both types. It has been suggested that increasing the positive charge of AMPs acting against Gram-negative bacteria could promote their interaction with LPS [88] and that hydrophobic residues are determinant for ensuring that these bacteria’s internal membranes collapse but not for permeabilising and crossing the external membrane [89]. However, increasing the charge can lead to reduced activity against Gram-negative membranes when helical peptides’ polar face hydrophobic amino acids are substituted [90]. The above shows the difficulty in finding patterns or direct relationships between an AMP’s physicochemical properties and its activity regarding one or other types of bacteria; such difficulty arises from the countless possible combinations regarding physicochemical characteristics making a particular peptide active and selective and the particularity of the interactions occurring between a specific AMP and a microorganism [18].

The forgoing has led to suggesting the need for insisting on two criteria when modifying peptides to focus their selectivity for some type of bacteria. Gram-positive and Gram-negative outer envelope and each peptide’s behaviour must be considered when interacting with the membranes of both types of bacteria; methods for modelling peptides’ interactions with membranes appear to be a key point in achieving this. For example, Liscano et al., used modelling to suggest that helical AMP alyteserin-1c structural stability may differ according to Gram-negative or Gram-positive membrane environment and that such helical stability is essential for activity against bacteria [90].

## 5. Challenges and Perspectives

The search for new effective anti-Gram-negative bacteria antimicrobials involves significant chemical and biological challenges [91]; microorganism-selective AMPs are thus extremely valuable in the search for and optimisation of new anti-Gram-negative bacteria therapeutic agents since they have breached one of the earliest barriers regarding AMPs, selectivity, even more so when it is known that a therapeutic agent’s window becomes reduced as it advances along the long road of clinical development [92]. Efforts from initial laboratory stage onwards must focus on candidates having very competitive characteristics regarding activity and selectivity.

Despite the approaches adopted by some research regarding the physicochemical characteristics defining AMP activity and selectivity, much still needs to be discerned regarding these AMPs ’interaction with Gram-negative bacterial envelop. New AMP design is a complex process requiring different research focuses backed by the use of computational tools.

Bioinformatics tools predicting generalised antimicrobial activity have supported AMP design for some time now; however, as time has passed, some authors have suggested the need for developing AMPs having activity against specific pathogens so as not to affect a host’s normal microbiota and ensuring a finer approach to AMP design [93,94]. Enormous efforts have been made at identifying characteristics differentiating AMP selectivity for determined bacteria, though much remains to be understood [87,88,89]. Many biophysical approaches, such as isothermal titration calorimetry (ITC), bioinformatics simulations and NMR, have broadened precise knowledge regarding key interactions for AMP action, laying down guidelines for future design [95,96,97,98,99,100,101].

Some activity prediction tools are undoubtedly essential as the first filter for designing and selecting AMPs. The Universidad Industrial de Santander in Colombia has developed in silico tools such as DEPRAMPs software; this is based on a genetic algorithm and is currently being patented. Prada et al., used this algorithm to design the 17 residue-long GIBIMPY4 (SFIKRSLKLIKSLVLIK) peptide which is active against pathogenic bacteria such as *E. coli* O157:H7 and methicillin-resistant *S. aureus*, but has a considerable cytotoxic and haemolytic effect [102]. Other peptides having great antibacterial potential but low selectivity (haemolytic and cytotoxic effect) have been designed using this software [103], thereby highlighting the need to complement the design algorithm method with haemolytic and/or cytotoxic activity prediction for obtaining AMPs which are highly selective for bacterial cells.

The DBAASP website (https://dbaasp.org, accessed on 1 December 2021), enabling peptides’ antimicrobial activity to be predicted regarding microbial species such as *E. coli*, *S. aureus* and *C. albicans* is based on a machine learning algorithm and has provided promising results regarding new AMP design [104,105,106]. This website enables predicting a target peptide’s activity against human erythrocytes [104]. Tools like this will surely accelerate obtaining new therapeutic candidates having a high TI against Gram-negative bacteria.

It is worth stressing that even though AMPs seem to represent hope for combating Gram-negative bacteria, one of the greatest difficulties for developing them lies in LPS variation and such bacteria’s membrane PL composition. From a bioinformatics point of view, in silico approaches involving artificial intelligence, molecular dynamics, microorganism surface visualisation must be involved to refine predicting AMP–Gram-negative bacteria interactions. Other approaches concerned with indirect action against pathogens or combined therapies represent a promising field for research from an experimental point of view.

Three peptides in clinical development (AB103 (p2TA), SGX942 (dusquetide) and EA-230) have immunomodulatory effects which could help resolve infections in in vivo conditions (Table 1 and Figure 1). While these peptides lack direct activity against microorganisms, they can attenuate the inflammatory effect caused by bacteria and LPS and has been shown to reduce death in a murine model of sepsis affecting septic shock mechanisms [35,36]. Such an approach involving interaction with the immune system represents a field of research which has only just begun and will surely help in combating the development of Gram-negative bacteria resistance and that of other pathogens from a different perspective [34].

Combined antimicrobial therapies such as AMP–antibiotics, AMP–AMP, AMP–nanoparticles, AMP–adjuvants and even AMP–immunomodulatory peptide therapies are of interest for overcoming the challenges imposed by microorganisms’ constant evolution; such a combined approach might decelerate the development of resistance, thus providing a head start that could enable reducing the lag in developing new therapies against MDR Gram-negative bacteria, particularly regarding Gram-negative ESKAPE pathogens that have high intrinsic antibiotic resistance and can acquire additional resistance through mutations and horizontal gene transfer [107]. Such therapies could reduce a microorganism’s chances of survival, simultaneously affecting the enzymes mediating its variability and developing resistance against them, as well as neutralising adverse effects caused by the release of toxins like LPS. However, combined therapies imply an increase in experimental variables which no one yet knows how to deal with and pose a real challenge for the scientific community [108].

## 6. Conclusions

Gram-negative bacteria-related infection represents a growing public health problem and the scientific community has not been able to respond with the same speed as that for developing new antibiotics. Few AMPs against Gram-negative bacteria are in clinical stage or have been FDA approved; some have not undergone the necessary optimisation to allow their systemic application, thereby restricting them to topical use. This has shown the scientific community the need to improve in vitro research for the early optimisation of new AMP candidates. Launching a list of requirements for promoting AMPs to pre-clinical stages could reduce the amount of therapeutic failures during advanced development stages regarding FDA approval and would enable efforts to be concentrated on molecules having the greatest therapeutic potential. TI ≥ 2 and molecule stability in the presence of human serum should be considered as essential requirements.

Gram-negative bacteria membrane complexity poses significant challenges for developing new therapies; LPS and inner and outer membrane PL bilayer composition condition susceptibility or resistance to AMPs, thereby stressing the relevance of negative charge density as a key characteristic in AMP–membrane interaction. Positive charge, helicoid structure, hydrophobic amino acid content and the E, K, K/R, K, K/R, K/R, K/R motif should be taken into account when designing anti-Gram-negative AMPs. Studies should also concentrate on the in-depth study of Gram-negative bacteria envelope to help define AMP sensitivity and resistance and ascertain an AMP’s structural conformation when in contact with the bacterial envelope’s environment.

The complexity of the relationship between an AMP’s physicochemical characteristics and its activity and selectivity demands that experimental exploration be complemented with computer tools. The DBAASP website (https://dbaasp.org/prediction, accessed on 1 December 2021) is worth stressing as an innovative tool for developing anti-Gram-negative bacteria AMPs. Using AMPs in combination with other antimicrobial agents, adjuvants or immunomodulatory peptides could be an effective strategy for containing Gram-negative bacteria activity, thereby increasing currently available therapies’ scope.

## Figures and Tables

**Table 1:** FDA-approved peptides or those in development against Gram-negative bacteria [7,14,21].

AMPs against Gram-Negative Bacteria									
Status/ Clinical Phase	Peptide	Description	Target	In Vitro Gram- Negative MIC	In Vitro Gram- Positive MIC	In Vitro Haemolysis	MoA	Route	Ref.
FDA- approved	Colistin or polymyxin E (natural)	Cationic cyclic lipodecapeptide isolated from *Paenibacillus polymyxa var colistinus*	Gram-negative	≤2 µg/mL (≤1.7 μM)	-	0–1.8% at 0.12 µg/mL	Membranolytic	Top, oral, IV	[21,22,23,24,25]
Phase III	Pexiganan o MSI-78 (designed)	A 22 amino acid-long magainin cationic analogue	Pathogens associated with diabetic foot infection	8–16 µg/mL (3.23–6.46 µM)	8–32 µg/mL (3.23–12.9 µM)	5–63% at 50–64 µg/mL	Toroidal pore former	Top	[19,26,27,28,29]
Phase II	LL-37	hCAP18 human cathelicidin-derived peptide	Broad bacterial and fungi spectrum	0.2–72 µg/mL (0.04–16 µM)	0.7–72 µg/mL (0.16–16 µM)	1.5–5% in MIC range	Membranolytic, binding to LPS and Immune modulation	Top	[30,31,32]
**Immunomodulatory Peptides against Gram-Negative Bacteria**									
**Status**/ **Clinical Phase**	**Peptide**	**Description**	**Target**	**In Vitro Gram-Negative MIC**	**In Vitro Gram**- **Positive MIC**	**In Vitro** **Haemolysis**	**MoA**	**Route**	**Ref.**
Phase III	AB103 (D-ala-p2TA) (designed)	CD28 homodimer interface mimetic octapeptide (CD288–15) abutted with D-Ala at both termini	Effective in mice challenged with *E. coli* bacteria	Only acts in vivo	Only acts in vivo	-	Attenuates CD28 T-cell signalling in exacerbated immune responses during infection	IV	[33]
Phase III	Dusquetide (IMX942, SGX942) (designed)	Synthetic 5-amino acid-long peptide	Gram-negative (i.e., *Burkholderia* *pseudomallei*) and Gram-positive (i.e., *S. aureus*) bacterial infections	Only acts in vivo	Only acts in vivo	-	Innate defence regulator (IDR), complement for antibiotics	IV	[34]
Phase IIa	EA-230 (Peptide 46)	Human chorionic gonadotropin derivate tetrapeptide (AQGV)	Effective in cercal ligation and puncture (CLP) mouse model	Only acts in vivo	Only acts in vivo	-	Immune modulation	IV	[35,36]

MoA: Mechanism of action, intravenous (IV), topical (Top).

**Table 2:** AMPs in in vitro investigation against Gram-negative bacteria.

Family	ID	Sequence	Target/MIC	Ref
Cathelicidins (LL-37)	KR-12-a5 (α-helix)	KRIVKLILKWLR-NH_2_	Both Gram-positive and Gram-negative bacteria/2–8 µM	[52]
Defensins	HD5d5	ARARCRRGRAARRRR LRGVCRIRGRLRRLAAR	*A. baumannii/* 40 µg/mL (10.4 µM)	[53]
Histatins	P-113D (α-helix)	d-AKRHHGYKRKFH-NH_2_	Both Gram-positive, Gram-negative and *Candida/* *P. aeruginosa*: 2 µg/mL (1.28 µM)	[54]
Magainins	Mag2 peptide 2	H-S5IKKS5LKSAKKFVKAFK-NH_2_	Both Gram-positive and Gram-negative bacteria/Gram-negative: 1.56–3.1 µM	[55]
Protegrins	L27-11 (β-sheet)	TWLKKRRWKKAK	*Pseudomonas* spp./ 0.004–0.01 µg/mL (0.0026–0.0064 µM)	[56]
Bacteriocins	Microcin J25 (MccJ25) Recombinant (β-sheet)	GGAGHVPEYFVGIGTPISFYG	Gram-negative/3.2–10.6 µg/mL (1.5–5 µM)	[57]
Cecropins	DAN2 (α-helix)	RWKFLKKIEKVGRKVRDGVIKAGPAVGVVGQATSIYK-NH2	Gram-negative/2–16 µg/mL (0.49–3.92 µM)	[58]

## Data Availability

Not applicable.
